# Supplementary material for: Cilia-enriched oxysterol 7β,27-DHC is required for polycystin ion channel activation
Source: Nat Commun. 2024 Jul 31;15:6468. doi: 10.1038/s41467-024-50318-9 (PMC11291729; doi:10.1038/s41467-024-50318-9)
Supplement: Supplementary file 1 — Supplementary Information [file 41467_2024_50318_MOESM1_ESM.pdf]

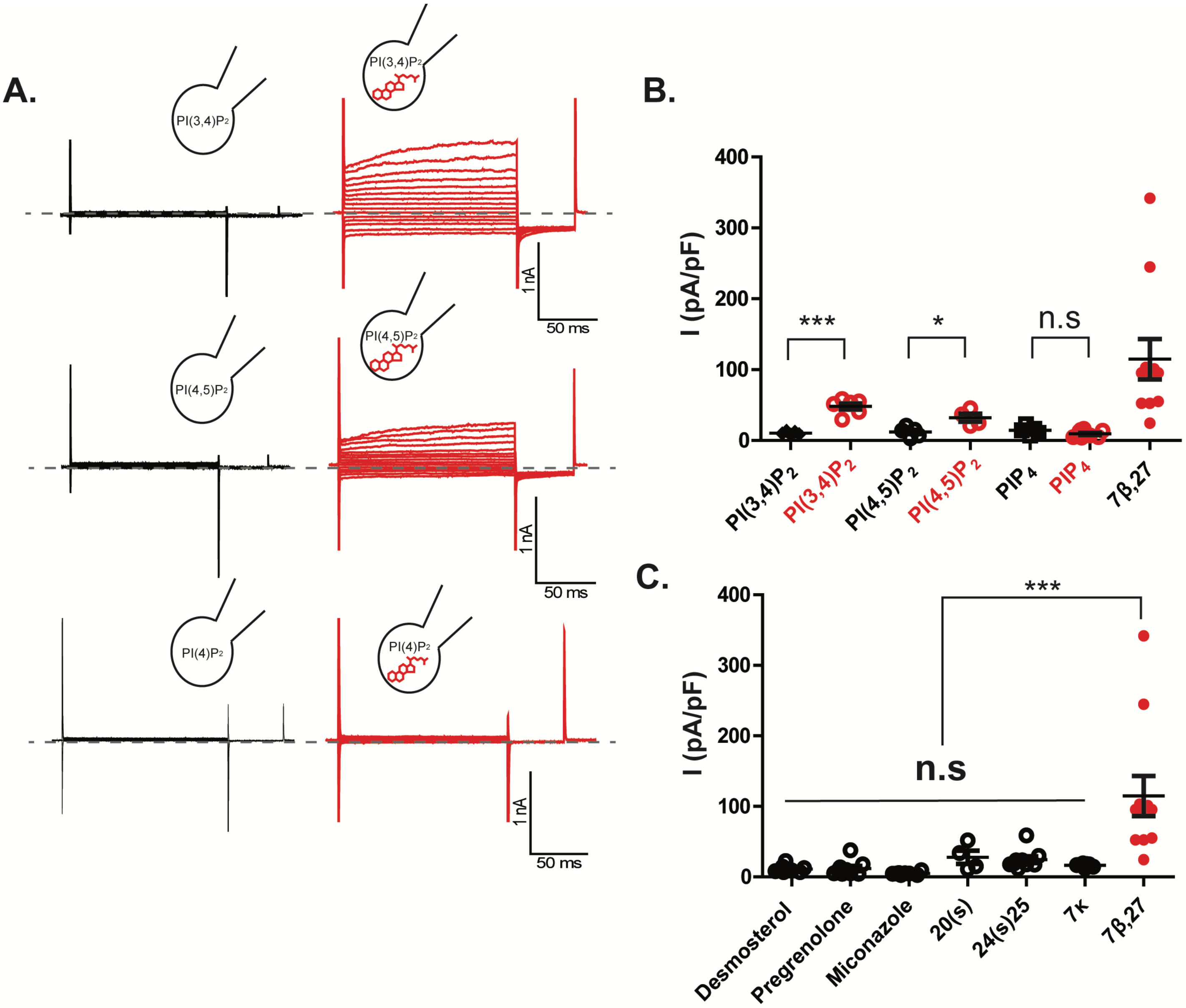

Supplementary Figure 1. Phosphatidylinositides compete with 7β,27-DHC.

A. Representative whole cell recordings of sPC-1/PC-2 with 5μM of PI(3,4)P_2, PI(4,5)P_2, and PI(4)P inside the pipette. Left. Pipette solution contains only phosphatidylinosides. Right. Pipette contains indicated phosphatidylinositide together with 5μM 7β,27-DHC. B. Average current density of sPC-1/PC-2 with phosphatidylinositides or phosphatidylinositides and 7β,27-DHC in the intracellular solution at +180 mV. PI(3,4)P_2 (n=5), PI(4,5)P_2 (n=5), PI(4)P (n=5). PI(3,4)P_2+7β,27-DHC (n=6), PI(4,5)P_2+7β,27-DHC (n=4), PI(4)P+7β,27-DHC (n=10). Two-tailed student's t-test. ***P<0.001, **P<0.01. C. Average current density of cholesterol derivatives and oxysterols all applied at 5μM within pipette. Desmosterol (n=10), pregnenolone (n=9), miconazole (n=8), 20(s) (n=5), 24(s)25 (n=8), 7k (n=7). Current amplitudes at +180 mV were obtained for the average current density (pA/pF). 5μM working concentration was used for all chemical compounds. Two-tailed student's t-test. ***P<0.001. Data represented as mean ± S.E.M.

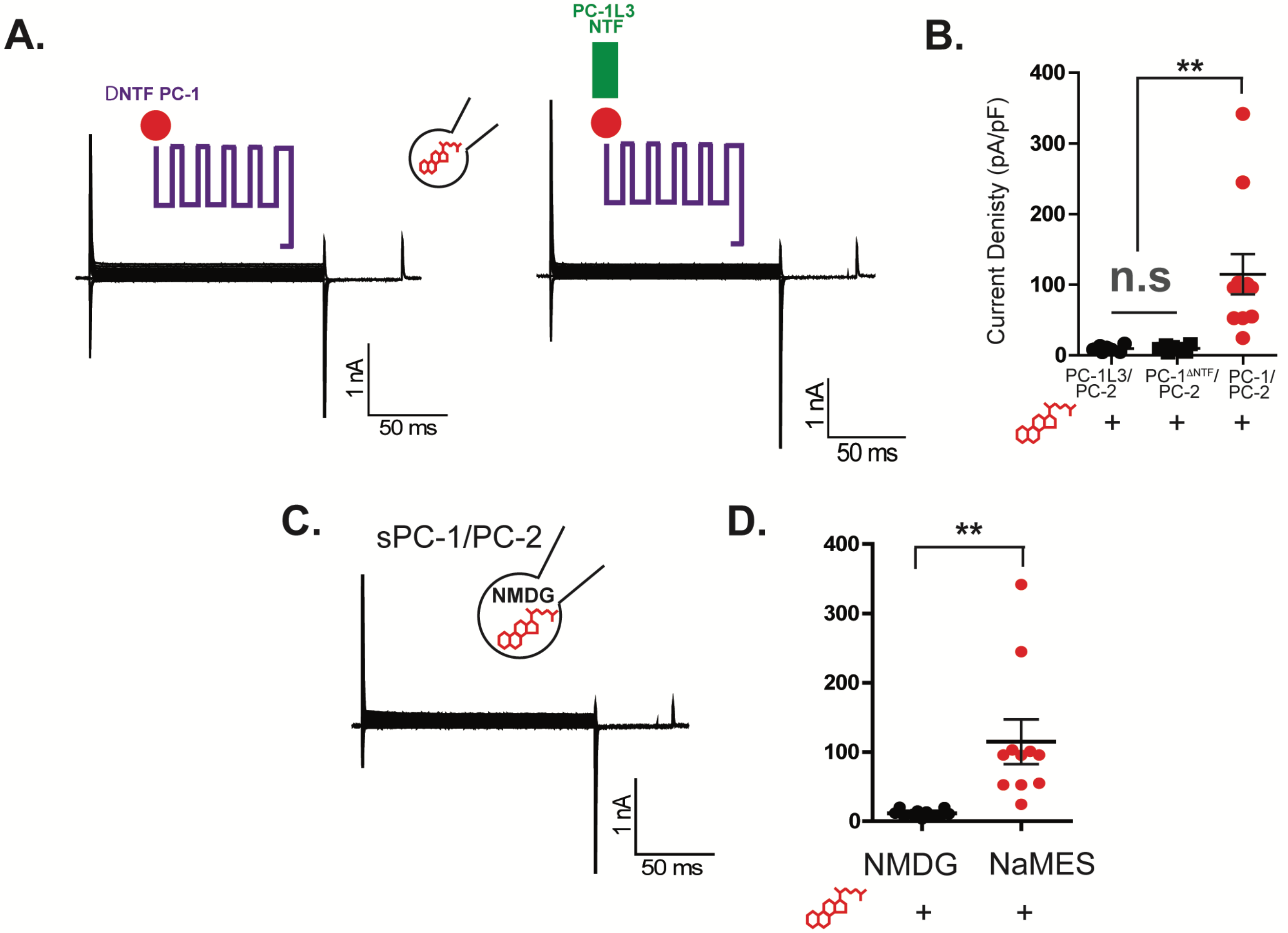

Supplementary Figure 2. 7β,27-DHC only activates full length sPC-1/PC-2 but not sPC1-L3/ PC-2 complex.

A. Whole-cell patch clamp recordings of HEK293 cells overexpressing PC-1$^{\Delta NTF}$/PC-2 or sPC-1L3/PC-2 cells. B. Average current density obtained at +180 mV from sPC-1/PC-2 (n=11), $^{\Delta NTF}$ PC-1/PC-2 (n=8), and sPC-1L3/PC-2 (n=8) with intracellular application of 5 μM 7β,27-DHC. Two-tailed unpaired student's t-test. **P<0.01. C. Whole-cell patch clamp recordings from sPC-1/ PC-2 with intracellular application of 5 μM 7β,27-DHC and NMDG. D. Average current density obtained at +180 mV from sPC-1/PC-2 with intracellular application of sodium methanesufonate (n=11) and NMDG (n=11). Two-tailed Student's t-test. ** P<0.01. Data represented as mean ± S.E.M.

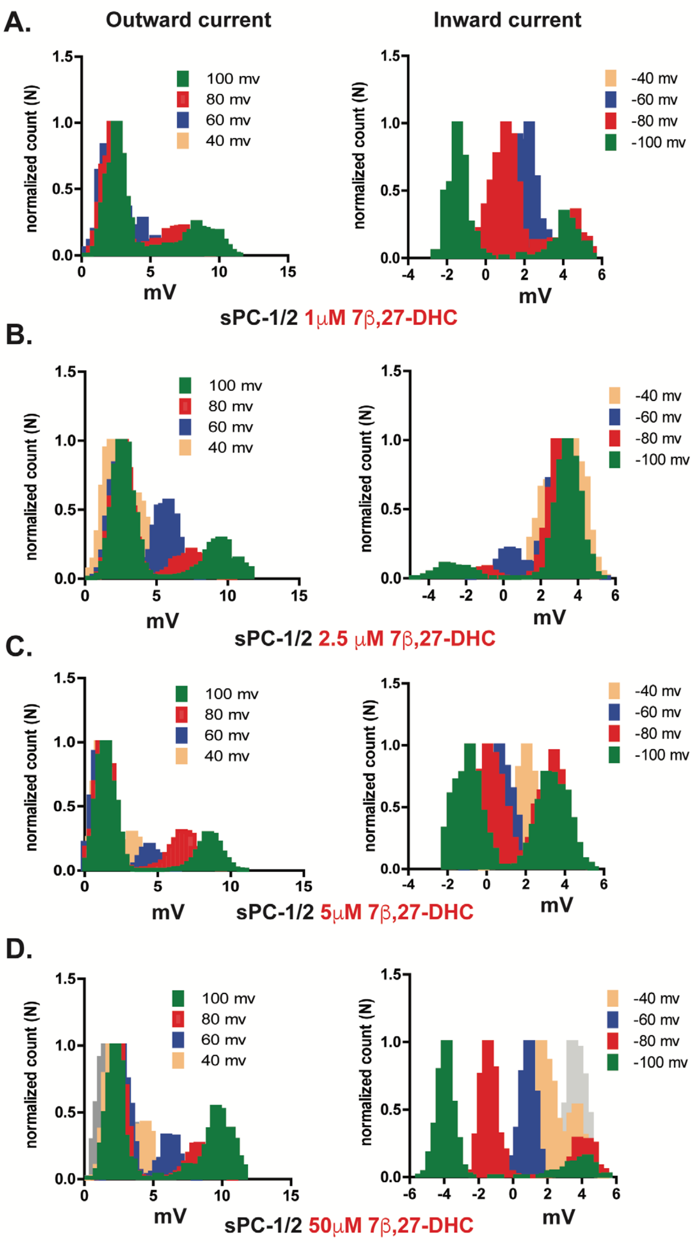

Supplementary Figure 3. Representative histogram collecting open channel events from the dose-dependent experiment of figure 2.

A-D. All-points histograms collecting open channel events of the inside-out single channel recordings of Figure 2. Currents were normalized to the maximum currents. Histograms collected from outward amplitudes (left) and inward amplitudes (right) were plotted from -20 mV to +100 mV.

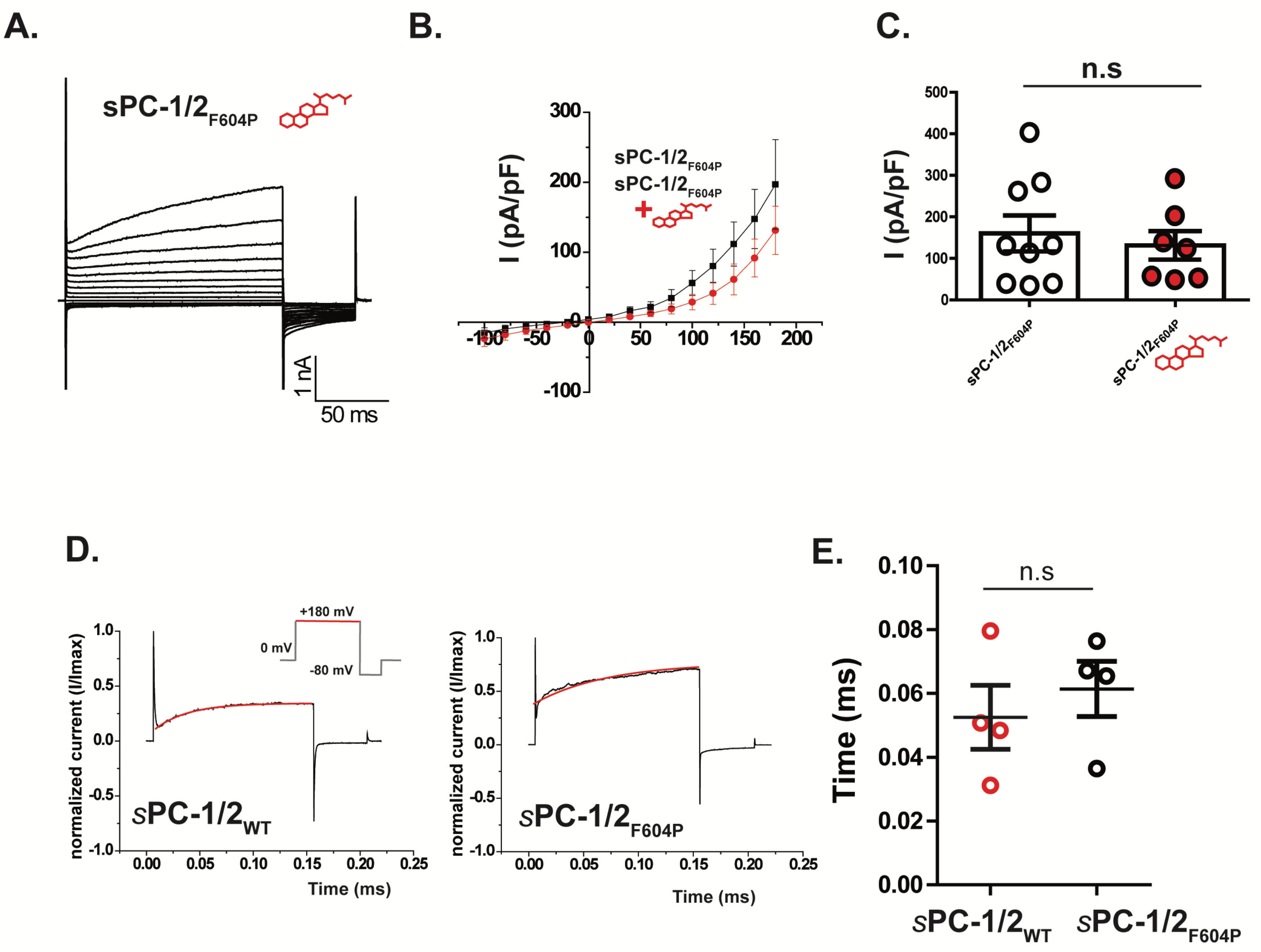

Supplementary figure 4. 7β,27-DHC does not further potentiate the GOF mutant, PC-2_{F604P}.

A. Representative whole-cell patch-clamp recording of sPC-$1/2_{F604P}$ with 7β,27-DHC. B. I-V relation of sPC-1/2 and sPC-$1/2_{F604P}$ with the 5μM 7β,27-DHC intracellular treatment; sPC-$1/2_{F604P}$ (n=9), sPC-$1/2_{F604P}$ with 5μM 7β,27-DHC (n=7). C. Comparison of the current density from Supplementary figure 2B at+180 mV membrane potential; sPC-$1/2_{F604P}$ (n=9), sPC-$1/2_{F604P}$ with 5μM 7β,27-DHC (n=7). All summary data, mean ± SEM. D. Comparison of activation kinetics elicited by a +180 mV voltage pulse for 150 ms. The currents at +180 mV of sPC-$1/2_{WT}$ and sPC-$1/2_{F604P}$ were fitted to the exponential. E. The fitted time constant (tau) of sPC-$1/2_{WT}$ (red) and sPC-$1/2_{F604P}$ (black). n=4, two-tailed Student's t-test. Data represented as mean ± S.E.M.

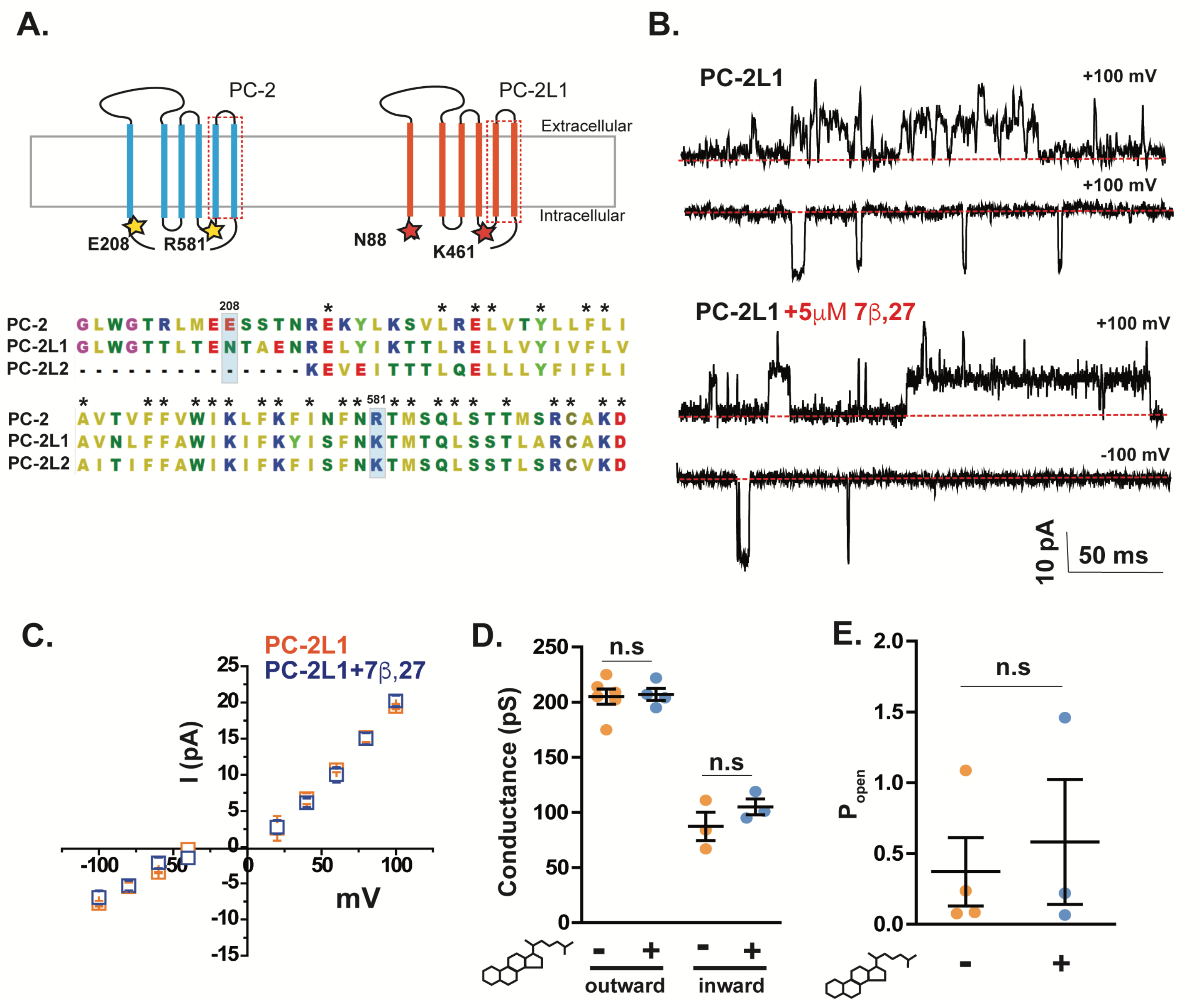

Supplementary figure 5. Oxysterol binding pocket is only present in PC-2.

A. Schematic diagram of PC-2 and PC-2L1 and protein alignment of putative oxysterol binding region in PC-2 with corresponding region in PC-2L1 and PC-2L2. Yellow stars indicate the location of oxysterol-binding mutants, E208 and R581, on the topology of PC-2. Red stars indicate N88 and K491, the location of corresponding oxysterol-binding sites in PC-2L1. A black star on the protein sequences indicates 100% conserved amino acids among PC-2 family proteins. B. Representative single-channel recordings of PC-2L1 at -100 mV and +100 mV with (bottom) and without (top) intracellular 5μM 7β,27-DHC. Red dotted line indicates the closed channel state during recordings. C. Current amplitudes collected from -100 mV to + 100 mV. Data are fitted to the linear equation to calculate conductance (γ). Blue and orange dotted lines indicate the linear fitting to the average current amplitudes of PC-2L1 with (blue, n=6) or without (orange, n=5) intracellular 5μM 7β,27-DHC. D. Comparison of PC-2L1 conductance obtained from inward and outward currents with (blue, outward n=6; inward n=3) or without (orange, outward n=4; inward n=3) intracellular 5μM 7β,27-DHC. E. Comparison of PC-2L1 open probability at -100 mV with (blue, n=3) and without (orange, n=4) intracellular 5μM 7β,27-DHC. Data represented as mean ± S.E.M.

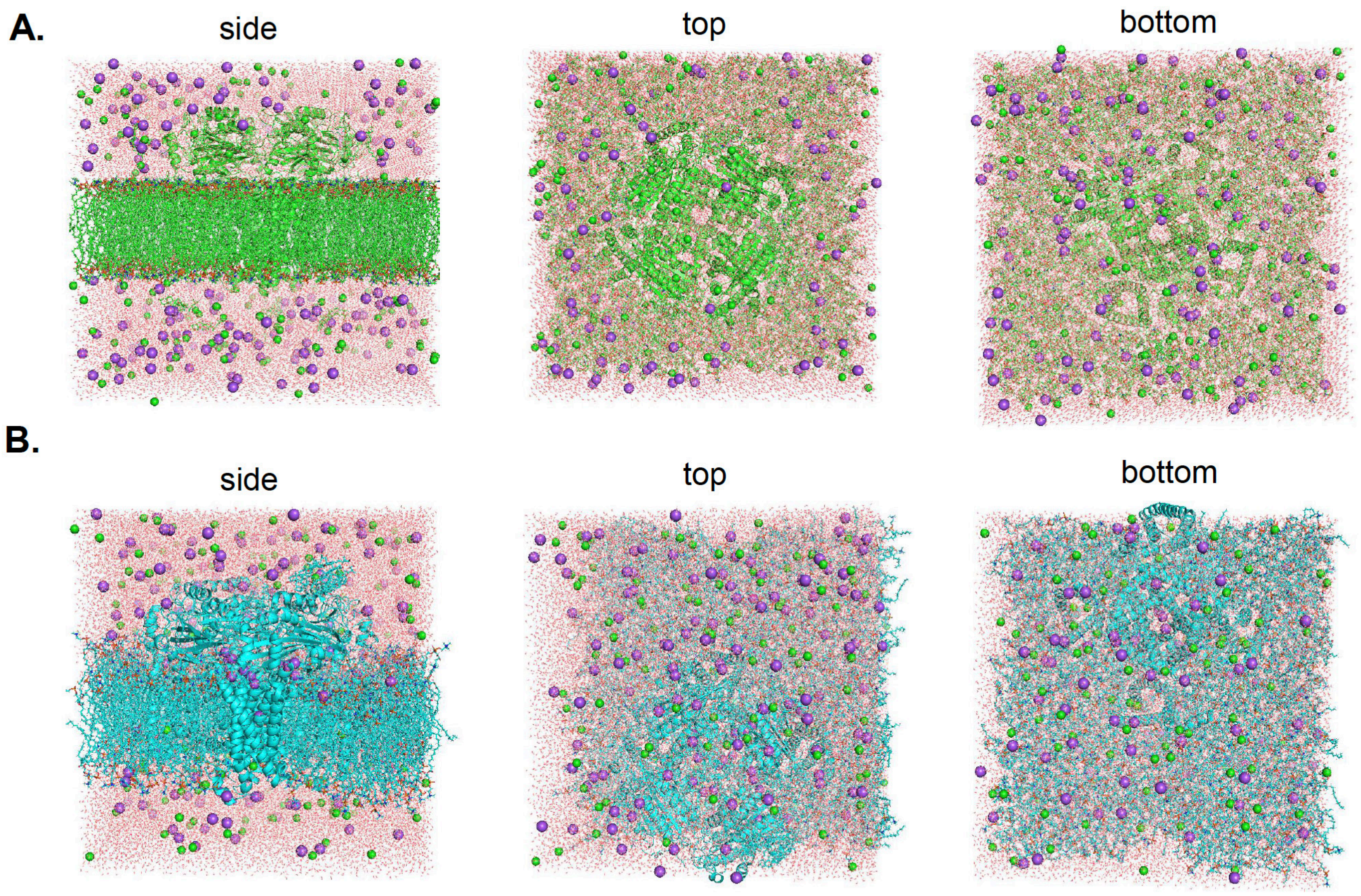

Supplementary figure 6. Snapshots of molecular dynamics trajectories shown in figure 3C and D.

A. Initial and B. final configurations for MD trajectories. Purple sphere: sodium; Green sphere: potassium.
