# Supplementary material for: Cilia-enriched oxysterol 7β,27-DHC is required for polycystin ion channel activation
Source: Nat Commun. 2024 Jul 31;15:6468. doi: 10.1038/s41467-024-50318-9 (PMC11291729; doi:10.1038/s41467-024-50318-9)
Supplement: Supplementary file 3 — Reporting Summary [file 41467_2024_50318_MOESM3_ESM.pdf]

nature portfolio

Corresponding author(s): Markus Delling

Last updated by author(s): 2024/05/25

Reporting Summary

Nature Portfolio wishes to improve the reproducibility of the work that we publish. This form provides structure for consistency and transparency in reporting. For further information on Nature Portfolio policies, see our Editorial Policies and the Editorial Policy Checklist.

Statistics

For all statistical analyses, confirm that the following items are present in the figure legend, table legend, main text, or Methods section.

n/a	Confirmed	
☐	☒	The exact sample size (*n*) for each experimental group/condition, given as a discrete number and unit of measurement
☐	☒	A statement on whether measurements were taken from distinct samples or whether the same sample was measured repeatedly
☐	☒	The statistical test(s) used AND whether they are one- or two-sided<br>*Only common tests should be described solely by name; describe more complex techniques in the Methods section.*
☒	☐	A description of all covariates tested
☒	☐	A description of any assumptions or corrections, such as tests of normality and adjustment for multiple comparisons
☐	☒	A full description of the statistical parameters including central tendency (e.g. means) or other basic estimates (e.g. regression coefficient) AND variation (e.g. standard deviation) or associated estimates of uncertainty (e.g. confidence intervals)
☐	☒	For null hypothesis testing, the test statistic (e.g. *F*, *t*, *r*) with confidence intervals, effect sizes, degrees of freedom and *P* value noted<br>*Give P values as exact values whenever suitable.*
☒	☐	For Bayesian analysis, information on the choice of priors and Markov chain Monte Carlo settings
☒	☐	For hierarchical and complex designs, identification of the appropriate level for tests and full reporting of outcomes
☒	☐	Estimates of effect sizes (e.g. Cohen's *d*, Pearson's *r*), indicating how they were calculated

Our web collection on statistics for biologists contains articles on many of the points above.

Software and code

Policy information about availability of computer code

Data collection	Commercially available hardware (200B amplifier together with 1324A Digidata, Molecular Device, Axon Instruments) and software (Clampex 10.0, Molecular Device, Axon Instruments) was used for electrophysiological recordings.
Data analysis	We used commercially available software for electrophysiological analysis (Clampfit 10.0, Molecular Device, Axon Instruments) and publicly available software for molecular modeling (AmberTools, SHAKE algorithm and PMEMD engine). Further, a detailed description of modeling parameters is described in the methods section. ImageJ 1.51h, Prism 9

For manuscripts utilizing custom algorithms or software that are central to the research but not yet described in published literature, software must be made available to editors and reviewers. We strongly encourage code deposition in a community repository (e.g. GitHub). See the Nature Portfolio guidelines for submitting code & software for further information.

Data

Policy information about availability of data

All manuscripts must include a data availability statement. This statement should provide the following information, where applicable:

- Accession codes, unique identifiers, or web links for publicly available datasets
- A description of any restrictions on data availability
- For clinical datasets or third party data, please ensure that the statement adheres to our policy

Data supporting the findings of this paper are available from the corresponding author upon request.

Research involving human participants, their data, or biological material

Policy information about studies with human participants or human data. See also policy information about sex, gender (identity/presentation), and sexual orientation and race, ethnicity and racism.

Reporting on sex and gender	N/A
Reporting on race, ethnicity, or other socially relevant groupings	N/A
Population characteristics	N/A
Recruitment	N/A
Ethics oversight	N/A

Note that full information on the approval of the study protocol must also be provided in the manuscript.

Field-specific reporting

Please select the one below that is the best fit for your research. If you are not sure, read the appropriate sections before making your selection.

☒ Life sciences ☐ Behavioural & social sciences ☐ Ecological, evolutionary & environmental sciences

For a reference copy of the document with all sections, see nature.com/documents/nr-reporting-summary-flat.pdf

Life sciences study design

All studies must disclose on these points even when the disclosure is negative.

Sample size	At least 10 cells were patched for each mutant or condition, a well-established sample size for electrophysiological recordings
Data exclusions	No Data excluded
Replication	all experiments were performed at least in three biological replicates and included in data analysis
Randomization	Control and experimental samples were analyzed alternately
Blinding	polycystin mutants we tested blinded to the experimenter

Reporting for specific materials, systems and methods

We require information from authors about some types of materials, experimental systems and methods used in many studies. Here, indicate whether each material, system or method listed is relevant to your study. If you are not sure if a list item applies to your research, read the appropriate section before selecting a response.

Materials & experimental systems

n/a	Involved in the study	
☐	☒	Antibodies
☐	☒	Eukaryotic cell lines
☒	☐	Palaeontology and archaeology
☒	☐	Animals and other organisms
☒	☐	Clinical data
☒	☐	Dual use research of concern
☒	☐	Plants

Methods

n/a	Involved in the study	
☒	☐	ChIP-seq
☒	☐	Flow cytometry
☒	☐	MRI-based neuroimaging

Antibodies

Antibodies used	rat anti-HA (3F10, Roche), mouse anti-Arl13b (N295B/66, Antibodies Incorporated), rabbit anti-HA (C29F4, Cell Signaling), mouse anti PKD2 (D3, Santa Cruz Biotechnology)
Validation	antibodies have been extensively characterized and and are frequently cited, according to manufacture's websites.

Eukaryotic cell lines

Policy information about cell lines and Sex and Gender in Research

Cell line source(s)	HEK293 and IMCD3 cells were obtained from ATCC.
Authentication	Not authenticated
Mycoplasma contamination	Cell lines tested negative for mycoplasma through through routine testing
Commonly misidentified lines (See ICLAC register)	These lines are not commonly misidentified

Plants

Seed stocks	N/A
Novel plant genotypes	N/A
Authentication	N/A
